# Primary and Metastatic Cutaneous Melanomas Discriminately Enrich Several Ligand-Receptor Interactions

**DOI:** 10.3390/life13010180

**Published:** 2023-01-08

**Authors:** Michael J. Diaz, Angela Fadil, Jasmine T. Tran, Sai Batchu, Kevin T. Root, Andrew X. Tran, Brandon Lucke-Wold

**Affiliations:** 1 College of Medicine, University of Florida, Gainesville, FL 32610, USA; 2 School of Medicine, University of Indiana, Indianapolis, IN 46202, USA; 3 Cooper Medical School, Rowan University, Camden, NJ 08103, USA; 4 Department of Dermatology, Case Western Reserve University School of Medicine, Cleveland, OH 44106, USA; 5 Department of Neurosurgery, University of Florida, Gainesville, FL 32611, USA

**Keywords:** cutaneous melanoma, interactome, ligand-receptor, transcriptome, omics

## Abstract

Introduction: Cutaneous melanoma remains a leading cancer with sobering post-metastasis mortality rates. To date, the ligand-receptor interactome of melanomas remains weakly studied despite applicability to anti-cancer drug discovery. Here we leverage established crosstalk methodologies to characterize important ligand-receptor pairs in primary and metastatic cutaneous melanoma. Methods: Bulk transcriptomic data, representing 470 cutaneous melanoma samples, was retrieved from the Broad Genome Data Analysis Center Firehose portal. Tumor and stroma compartments were computationally derived as a function of tumor purity estimates. Identification of preferential ligand-receptor interactions was achieved by relative crosstalk scoring of 1380 previously established pairs. Results: Metastatic cutaneous melanoma uniquely enriched PTH2-PTH1R for tumor-to-stroma signaling. The Human R-spondin ligand family was involved in 4 of the 15 top-scoring stroma-to-tumor interactions. Receptor ACVR2B was involved in 3 of the 15 top-scoring tumor-to-tumor interactions. Conclusions: Numerous gene-level differences in ligand-receptor crosstalk between primary and metastatic cutaneous melanomas. Further investigation of notable pairings is warranted.

## 1. Introduction

Cutaneous melanoma (CM) remains one of the most aggressive and deadliest forms of skin cancer, with epidemiological studies depicting the last few decades witnessing dramatic increases in incidences worldwide, with over 200,000 new cases diagnosed each year [1,2]. However, the morbidity and mortality rates of CM globally vary significantly, contingent on the availability of early screening and primary care [3]. This skin malignant tumor exhibits high metastasis, often leading to poor prognosis, as patients diagnosed with late-stage CM face a 5-year survival rate of less than 10% [4]. After surgical resection of the primary tumor, approximately ⅓ patients with CM experience metastasis in other organs at a higher rate than other forms of skin cancers [5]. Despite significant efforts dedicated to managing advanced and metastatic CM, clinical management and therapeutics remain far less effective [2]. The pathogenesis and molecular biological indicators of CM are not completely clear [6]. Therefore, understanding the underlying molecular mechanisms of the growth, progression, and metastasis of CM may offer insight into developing novel therapeutics and improving the prognosis of advanced melanoma.

Bulk RNA sequencing (RNA-seq) provides extensive utilities for the classification of cancer, identification of biomarkers, disease diagnosis, and the optimization of treatments [7]. In the context of skin cancer, bulk RNA-seq-based approaches have been employed to identify novel genes, predict treatment response, propose new therapeutic targets, and facilitate the understanding of the progression and pathogenesis of a disease [8]. For example, RNA-seq illustrated novel targets and signaling pathways in basal cell carcinoma (BCC) that offered implications for new therapeutic targets [9]. For clarification in the molecular basis of skin malignancies, Chitsazzadeh et al. used RNA-seq to illustrate the dysplastic states on the cutaneous squamous cell carcinomas that signified the transcription factors involved in regulating their development [10]. RNA-seq revealed different transcriptome signatures that defined the two distinct routes for melanoma development in the early and late stages with implications for diagnostics and treatment of CM in an analysis of melanocytic nevi and primary melanomas performed by Kunz et al. [11]. Furthermore, targeted RNA-seq utilized by Svedman et al. identified novel genes and expression signatures that could serve as possible predictive biomarkers for targeted therapies and immunotherapies in patients with metastatic CM [12]. These aforementioned studies reflect how bulk RNA-seq-based analyses provided an invaluable foundation for examining the pathophysiological landscape of skin cancer.

The cellular composition of tumors consists of highly heterogenous tumor cells due to somatic genetic alterations and cells within the tumor microenvironment that results from the infiltration of the stroma and other immune cell types [7]. The spatiotemporal interactions between these components could pose a critical role in the pathogenesis of melanoma [13]. Exploring tumor cell populations and tumor-immune cell interactions inside a tumor in the context of ligand-receptor pairs could facilitate the development of immunotherapies and other effective targeted treatments [14]. Toward this end, bulk RNA profiles reveal cell-to-cell interactions, which can disclose distinct microenvironments associated with malignant cell profiles and how the subset of genes expressed by a single cell type can affect the proportion of cells in the presence of another cell type [13]. Though the raw sequencing output reveals only quantity of reads mapped to a known gene transcript, downstream deconvolution methods can utilize established gene signatures to estimate pre-specified cell type fractions [8]. One such specification is tumor (versus non-tumor), thereby generating distinct tumor and stroma expression compartments within a single tumor tissue sample. In the present study, we leverage a bulk deconvolution procedure to nominate important CM molecular interactions within these compartments. Since the manifestation and development of CM encompass mutual regulation by gene expression and the immune microenvironment, this study aims to elucidate the ligand-receptor signaling architecture in both primary tumors and metastasis from previously published bulk gene expression data.

## 2. Methods

Expression data. Bulk transcriptome data for primary and metastatic cutaneous melanomas were retrieved from the Broad GDAC Firehose portal (https://gdac.broadinstitute.org/, Accessed on 4 October 2022) (N = 470). These data are represented by The Cancer Genome Atlas (TCGA) effort.

Estimating tumor purity. The ESTIMATE algorithm was used to predict tumor purities, defined as the proportion of tumor cells in each tissue sample. ESTIMATE uses combined stromal and immune gene signature database to output “ESTIMATE scores”, which are convertible to purity estimates [15]. Expression data was normalized to TPM (transcripts per million) and log2-transformed prior to input.

Compartmental tumor and stroma gene expression. Tumor and stromal (nontumor) compartmental gene expression was expressed as previously described [16]. For a given gene in a bulk tumor sample, the total mRNA expression can be modeled as follows:

$$e_{bulk,i}=p_{i}{\bar{e}}_{T}+\left(1-p_{i}\right){\bar{e}}_{S}$$

$e_{bulk,i}$: bulk mRNA expression for given gene in tumor sample *i*

$p_{i}{\bar{e}}_{T}$: average mRNA expression level in the tumor compartment, where $p_{i}$ denotes tumor purity

$\left(1-p_{i}\right){\bar{e}}_{S}$: average mRNA expression level in the stromal compartment, where $\left(1-p_{i}\right)$ denotes the stromal fraction estimate.

Tumor and stroma compartment expression levels, defined in the above equation, were obtained via negative least-squares regression, with the assumption that these average compartment expression levels are constant across tumor samples. 95% confidence intervals for tumor and stroma point estimates were derived via bootstrapping.

Ligand-receptor interaction scoring. Compartmental expression values were annotated using a combined set of 1380 ligand-receptor pairs [16,17]. Applying the law of mass action, the molar concentration of a given ligand-receptor interaction complex can be modeled as follows:

$$\left[LR\right]=\left[L\right]\left[R\right]K_{D}^{-1}$$

[LR]: molar concentration of given ligand-receptor interaction complex

[L]: given ligand concentration

[R]: given receptor concentration

$K_{D}^{-1}$: dissociation constant for given ligand-receptor interaction complex

If the following conditions are assumed: (1) the inferred mRNA expression values are reasonable proxies for ligand and receptor concentrations, (2) ligand-receptor kinetics are constant across all samples, and (3) the law of mass action assumptions are met, then the following relative crosstalk (RC) score equation can be applied. Here, the numerator represents the ligand-receptor complex of interest, and the denominator represents all possible ligand-receptor complexes (or directionality). Example given below for tumor-stroma ligand-receptor interaction scoring.

$$RC_{T,S}=\frac{\left({\bar{e}}_{L,T}{\bar{e}}_{R,S}\right)K_{D}^{-1}}{({\bar{e}}_{L,T}{\bar{e}}_{R,S})K_{D}^{-1}+({\bar{e}}_{L,T}{\bar{e}}_{R,T})K_{D}^{-1}+({\bar{e}}_{L,S}{\bar{e}}_{R,S})K_{D}^{-1}+\left({\bar{e}}_{L,S}{\bar{e}}_{R,T}\right)K_{D}^{-1}}$$

$RC_{T,S}$: relative crosstalk score for tumor-stroma ligand-receptor interaction

${\bar{e}}_{L,T}{\bar{e}}_{R,S}$: interaction of tumor ligand and stromal receptor

${\bar{e}}_{L,T}{\bar{e}}_{R,T}$: interaction of tumor ligand and tumor receptor

${\bar{e}}_{L,S}{\bar{e}}_{R,S}$: interaction of stromal ligand and stromal receptor

${\bar{e}}_{L,S}{\bar{e}}_{R,T}$: interaction of stromal ligand and tumor receptor

Cancelling out the dissociation constant, the relative crosstalk score for a specified tumor ligand and stromal receptor pair, can be modeled by the following final equation:

$$RC_{T,S}=\frac{{\bar{e}}_{L,T}{\bar{e}}_{R,S}}{{\bar{e}}_{L,T}{\bar{e}}_{R,S}+{\bar{e}}_{L,T}{\bar{e}}_{R,T}+{\bar{e}}_{L,S}{\bar{e}}_{R,S}+{\bar{e}}_{L,S}{\bar{e}}_{R,T}}$$

Software. All analyses were conducted with Python (v3.10.7) and R (v.4.2.2). Initial data wrangling and figure creation was carried out with R (v.4.2.2).

## 3. Results

**Figure 1:**
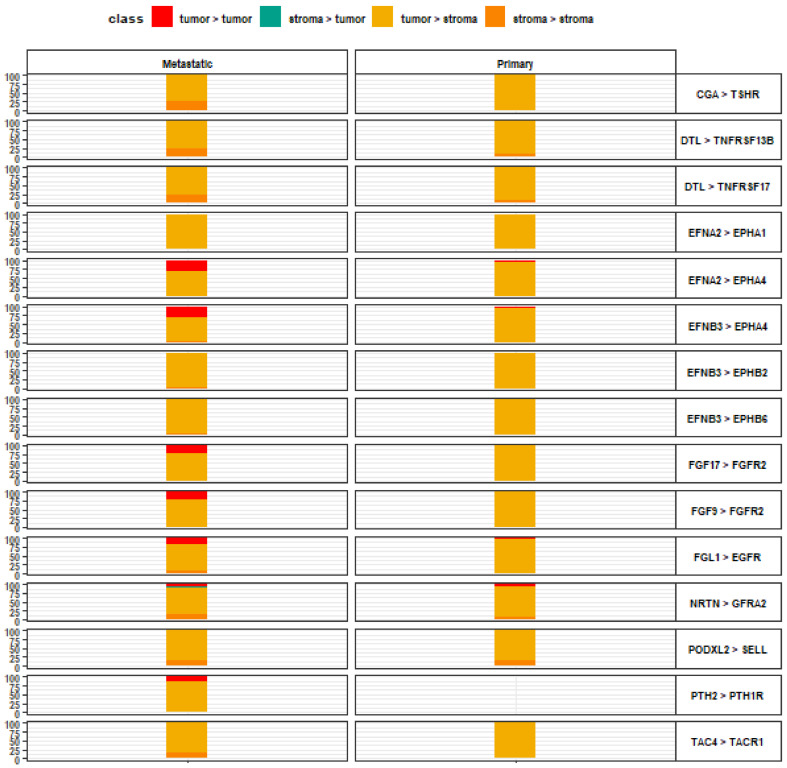
Top 15 ligand-receptor pairs with tumor-to-stroma signaling directionality. Leftmost axis represents directionality contributions (%) to the overall relative crosstalk score for a given ligand-receptor interaction complex. Rightmost axis describes the top-scoring ligand-receptor interaction complexes along the tumor-to-stroma signaling axis, wherein the left item denotes a tumor ligand and the right item denotes a stromal receptor, for each pair.

**Figure 2:**
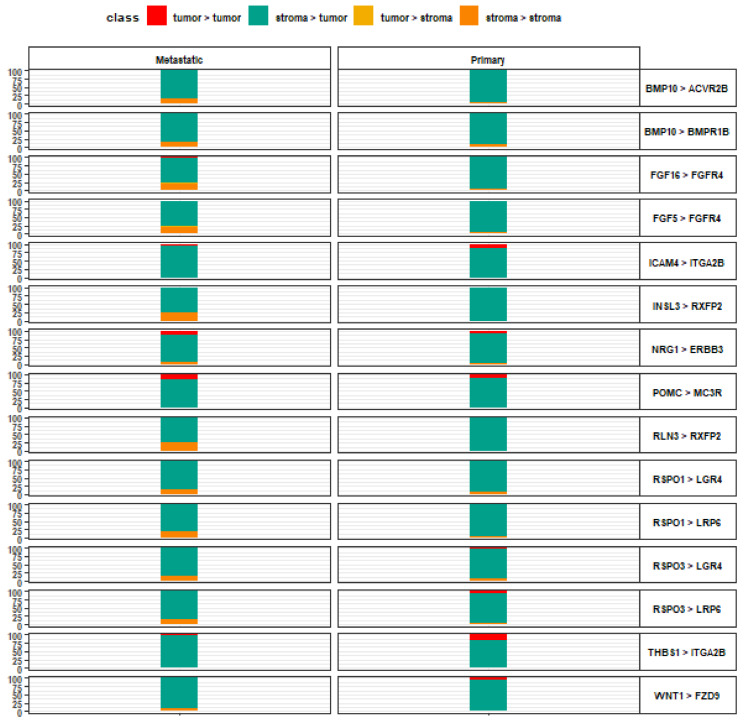
Top 15 ligand-receptor pairs with stroma-to-tumor signaling directionality. Leftmost axis represents directionality contributions (%) to the overall relative crosstalk score for a given ligand-receptor interaction complex. Rightmost axis describes the top-scoring ligand-receptor interaction complexes along the stroma-to-tumor signaling axis, wherein the left item denotes a stromal ligand and the right item denotes a tumor receptor, for each pair.

**Figure 3:**
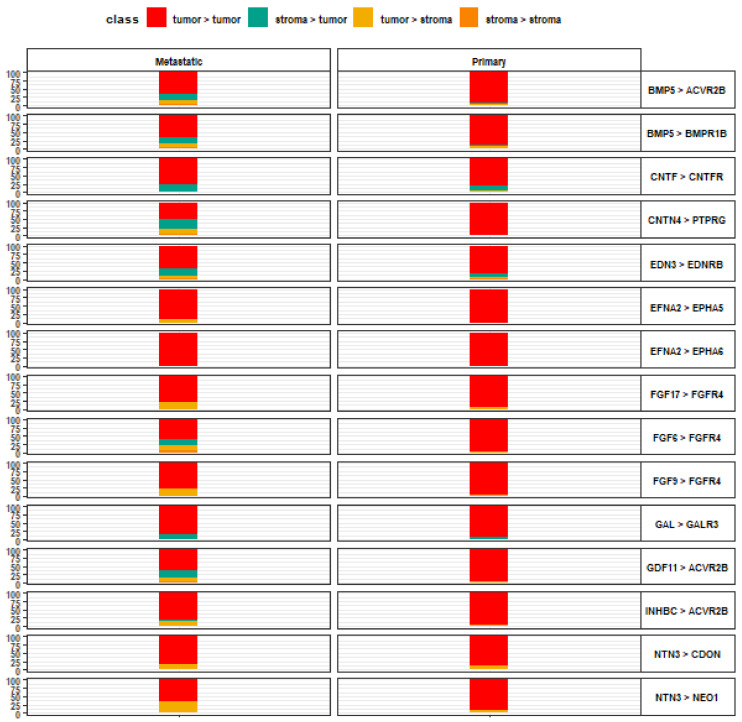
Top 15 ligand-receptor pairs with tumor-to-tumor signaling directionality. Leftmost axis represents directionality contributions (%) to the overall relative crosstalk score for a given ligand-receptor interaction complex. Rightmost axis describes the top-scoring ligand-receptor interaction complexes along the tumor-to-tumor signaling axis, wherein the left item denotes a tumor ligand and the right item denotes a tumor receptor, for each pair.

**Figure 4:**
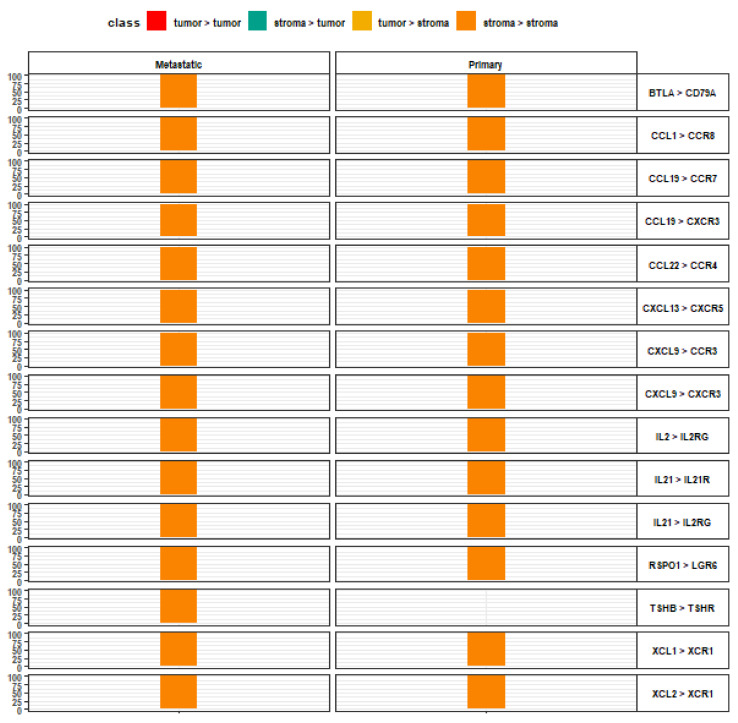
Top 15 ligand-receptor pairs with stroma-to-stroma signaling directionality. Leftmost axis represents directionality contributions (%) to the overall relative crosstalk score for a given ligand-receptor interaction complex. Rightmost axis describes the top-scoring ligand-receptor interaction complexes along the stroma-to-stroma signaling axis, wherein the left item denotes a stromal ligand and the right item denotes a stromal receptor, for each pair.

The highest-scoring ligand-receptor interactions in primary and metastatic lesions were evaluated for each signaling direction (Figure 1, Figure 2, Figure 3 and Figure 4). In tumor-to-stroma signaling, primary and metastatic lesions are characterized by EFNA2 interacting with EPHA1, EFNB3 interacting with EPHB2, and EFNB3 interacting with EPHB6, among several others (Figure 1). Metastatic lesions also exhibited PTH2-PTH1R interaction, which was not significant among primary lesions. Additionally, CGA-TSHR, EFNA2-EPHA1, FGF17-FGFR2, FGF3-FGFR2, and TAC4-TACR1 were high-scoring tumor-to-stroma signaling interactions in both primary and metastatic lesions. However, these interactions only decisively predominate in the tumor-to-stroma direction for primary lesions. Ephrin family ligand-receptor interactions accounted for 5 of the 15 to-scoring ligand-receptor interactions, indicating their importance in tumor-to-stroma signaling.

Ligand-receptor pairs with a preference for stroma-to-tumor signaling include BMP10-ACVR2B, FGF16-FGFR4, FGF5-FGFR4, INSL3-RXFP2, RLN3-RXFP2, RSPO1-LRP6, RSPO3-LGR4, and RSPO3-LRP6 (Figure 2). These same pairs have a lower stroma-to-tumor preference among metastatic lesions. In fact, the stroma-to-tumor direction is the only signaling direction without a top-scoring ligand-receptor interaction that consistently predominates in its direction among metastatic lesions. Also unique to this signaling direction, the overall highest-scoring ligand-receptor interactions are characterized by the predominance of Human R-spondin (RSPO) family ligands. RSPO proteins are involved in 4 of the 15 top-scoring ligand-receptor interactions.

Among the highest-scoring tumor-to-tumor interactions, EFNA2-EPHA6 was decisive in both primary and metastatic lesions (Figure 3). However, there were no further ligand-receptor interactions that were consistently specific for tumor-to-tumor interaction among metastatic lesions. Additional pairs with a strong preference for tumor-to-tumor signaling in primary lesions include CNTN4-PTPRG, EFNA2-EPHA5, FGF6-FGFR4, FGF9-FGFR4, GDF11-ACVR2B, and INHBC-ACVR2B. ACVR2B represents the common receptor in 3 of the 15 top-scoring tumor-to-tumor interactions.

Chemokine-receptor and interleukin-receptor pairs scored highly among stroma-to-stroma signaling (Figure 4). These interactions are not represented in the top 15 for any other direction. Other interactions found among both primary and metastatic lesions are BTLA-CD79A and RSPO1-LGR6. Among metastatic lesions only, stroma-to-stroma signaling also demonstrated TSHB-TSHR interactions. Furthermore, every identified stroma-to-stroma interaction is decisive in signaling direction. Each of the high-scoring ligand-receptor pairs displayed complete preference for stroma-to-stroma signaling. Additionally, stroma-to-stroma signaling is the only signaling direction that does not include fibroblast growth factor (FGF) family ligands in the top-fifteen signaling interactions (Figure S1).

## 4. Discussion

FGF ligand-receptor interactions were implicated across multiple signaling directions in cutaneous melanoma. Previously, FGF signaling dysregulation has been identified across many types of cancer, although the underlying mechanism is not well understood [18]. However, Seitz et al. demonstrated that elevated FGFR expression prognosticates poor survival in uveal melanoma patients [19]. Notably, the FGF9 ligand specifically enhanced uveal melanoma proliferation. Furthermore, the results of our analysis also uncovered FGF9-FGFR4 as one of the highest-scoring ligand-receptor interactions in cutaneous melanoma. Past literature established homeostatic FGF-FGFR signaling as participating in skin surface expansion and protection from ultraviolet damage [18]. Further, FGF is associated with tumor growth, invasion, migration, angiogenesis, and FGF-FGFR signaling contributes to therapeutic resistance in melanoma [18,20]. Currently, the added downregulation of various FGF signaling pathways have been introduced as a therapeutic strategy demonstrating the significance of our findings. The ongoing LOGIC-2 clinical trial specifically evaluates targeting this pathway in resistant melanoma [20].

In our study, RSPO1 and RSPO3 were identified as important stroma-to-tumor ligands, each interacting with both the LGR4 and LRP6 receptors in cutaneous melanoma. The role of RSPO proteins in cancer has been largely derived from studies in the intestinal tract [21]. However, Tan et al. examined the role of RSPO-LGR4 signaling in murine melanoma model [22]. The LGR4/5/6 receptors of RSPO ligands are often expressed by stem cells and progenitor cells [21]. Blocking this signaling pathway restricted tumor growth and reversed resistance to anti-PD1 therapy [22]. These findings highlight the potential therapeutic value of RSPO-LGR4 signaling modification to improve tumor immunity. Notably, WNT1-FZD9 signaling was also identified in the stroma-to-tumor direction. This interaction is potentiated by RSPO proteins interacting with LRP5/6 receptors, which increase cell sensitivity to WNT ligands by upregulating cell-surface receptors [23]. The WNT1 ligand is associated with the canonical pathway, which is dependent on β-catenin to promote melanocyte differentiation and proliferation [24]. The practicality of targeting this pathway for melanoma treatment is unclear due to its complexity of influences and effects. For example, while increased WNT activity is associated with tumorigenesis, WNT-signaling is also related to cancer immunity [25]. Additionally, crosstalk with other signaling pathways influences the role of WNT-signaling [25]. Therefore, despite its promise, more studies elucidating this pathway are needed.

In our study, EFNA2-EPHA interactions scored highly among the tumor-to-tumor and tumor-to-stroma signaling directions. While dysregulation of EFN signaling has been shown to increase susceptibility to skin carcinogenesis [26], EFN-EPH signaling can exhibit both tumor suppressor and oncogene activities in various contexts [27]. The common functions of EFN subfamily EFNA are to promote keratinocyte differentiation, terminate proliferation, and inhibit migration specifically regulates ubiquitin-associated proteolysis genes and suppresses apoptosis and cell cycle genes [27]. While EFNA2 signaling modification has not been evaluated in cutaneous melanoma, upregulation of EFNA2 expression in gastric cancer tissue suggests a potential role in cancer behavior [28]. Additionally, Zhao et al. demonstrated that EFNA2 participates in the angiogenesis and epithelial-mesenchymal transformation of prostate cancer, further implying its tumorigenic actions [29].

In the tumor-to-tumor signaling direction, activin receptor type-2B (ACVR2B) was implicated in three of the fifteen top-scoring interactions with corresponding ligands of bone morphogenetic protein 5 (BMP5), growth differentiation factor 11 (GDF11), and inhibin beta C chain (INHBC). In the stroma-to-tumor signaling pathway, BMP5-ACVR2B signaling was highlighted as well. All three ligands belong to the transforming growth factor-beta (TGF-β) superfamily, in which increased expression has been associated with cancer cachexia [30]. Barreto et al. found that inhibition of ACVR2B signaling with a soluble ACVR2B fusion protein has been shown to counteract chemotherapy-induced muscle and bone loss [31]. Similarly, Huot and colleagues reported that ACVR2B inactivation prevented multi-organ perturbations secondary to cachexia in metastatic colon cancer [32]. The implications of the TGF-β superfamily extend beyond cachexia; the various superfamily ligands converge at the SMAD pathway, which controls hundreds of genes [33]. Broadly targeting the downstream effects with small molecule inhibitors that decrease SMAD phosphorylation (galunisertib and vactosertib) has shown promising antitumor activity [33].

Our results further demonstrated stroma-to-stroma signaling as predominantly characterized by interleukin (IL) and chemokine signaling. This ligand-receptor signaling architecture has practical applications in melanoma treatment. Cytokines, including the interleukin IL-2 and IL-21 ligands identified in cutaneous melanoma, regulate innate and adaptive immunity [34]. High-dose IL-2 infusions have been approved for the treatment of metastatic melanoma since 1998 [34]. IL-21 use had also shown efficacy in metastatic melanoma [35], but direct administration of IL-21 is no longer a standard practice due to adverse inflammatory effects and a lack of consistent clinical data [34]. Regarding chemokines, which are cytokines with the ability to stimulate cell migration, metastatic dissemination of melanoma has been closely linked to expression of specific chemokine receptors [36]. Additionally, they overlap with some of the receptors identified in the stroma-to-stroma analysis: CCR4, CCR7, and CXCR3 [36]. Upregulation of chemokine receptors facilitates melanoma progression and metastasis, and the targeting of these pathways has long-term impacts on immune cell development [36]. Importantly, chemokine receptor blockers combined with anti-PD-1 or anti-CTLA-4 antibodies have improved patient survival in metastatic melanoma, a phenomenon, in part, elucidated by our findings [37].

A primary limitation of the present analysis is the use of average gene expression for each compartment and treatment as constant across CM samples. It is well understood that tumor and stromal cell populations are not evenly distributed in space, thereby rendering the CM microenvironment highly variable from sample to sample. Additional limitations include the lack of both a replicative dataset and association with clinical parameters (e.g., survival and Breslow depth). Still, this semi-supervised approach valuably identified numerous molecular pairs eligible for deeper investigation, potentially proposing novel therapeutic targets with specificity for the melanoma disease course.

## 5. Conclusions

Cutaneous melanoma continues to rank among the deadliest cancers, yet efforts to analyze key ligand-receptor interactions remain paltry at the time of writing. In the present study, we reported a first-use case of crosstalk scoring for transcriptome data derived from primary and metastatic CM samples. Notable findings include the following: enrichment for tumor ligand PTH2 interactions with stromal receptor PTH1R in metastatic lesions; enrichment for human R-spondin family proteins in stroma-to-tumor signaling; enrichment for tumor ligand EFNA2 interactions with stromal receptor EPHA6; enrichment for chemokine and interleukin stromal receptors interacting with stromal ligands. These data offer novel protein targets of interest and support genomic change in the post-metastasis CM microenvironment. Future interactome efforts should aim to build onto this work with greater power by incorporating microarray and single cell expression datasets.

## Data Availability

Expression data is publicly available at the Broad GDAC Firehose portal (https://gdac.broadinstitute.org/, Accessed on 4 October 2022).

## Supplementary Materials

The following supporting information can be downloaded at: https://www.mdpi.com/article/10.3390/life13010180/s1, Figure S1: Distribution of signaling interactions.
